# Molecular detection of colistin resistance genes (*mcr-1*, *mcr-2* and *mcr-3*) in nasal/oropharyngeal and anal/cloacal swabs from pigs and poultry

**DOI:** 10.1038/s41598-018-22084-4

**Published:** 2018-02-27

**Authors:** Jilei Zhang, Li Chen, Jiawei Wang, Afrah Kamal Yassin, Patrick Butaye, Patrick Kelly, Jiansen Gong, Weina Guo, Jing Li, Min Li, Feng Yang, Zhixing Feng, Ping Jiang, Chunlian Song, Yaoyao Wang, Jinfeng You, Yi Yang, Stuart Price, Kezong Qi, Yuan Kang, Chengming Wang

**Affiliations:** 1 Jiangsu Co-Innovation Center for Prevention and Control of Important Animal Infectious Diseases and Zoonoses, Yangzhou University College of Veterinary Medicine, Yangzhou, Jiangsu 225009 P.R. China; 2 Department of Food Hygiene and safety, Faculty of Public and Environmental Health, Khartoum University, Khartoum, Sudan; 3 Department of Biosciences, Ross University School of Veterinary Medicine, PO Box 334, Basseterre, Saint Kitts and Nevis; 4 Department of Pathology, Bacteriology and Poultry diseases, Faculty of Veterinary Medicine, Ghent University, Ghent, Belgium; 5 Poultry Institute, Chinese Academy of Agricultural Sciences, Yangzhou, Jiangsu China; 6 College of Animal Science, Anhui Science and Technology University, Bengbu, 230001 China; 7 Institute of Veterinary Medicine, Jiangsu Academy of Agricultural Sciences, Key Laboratory of Veterinary Biological Engineering and Technology, Ministry of Agriculture, National Center for Engineering Research of Veterinary Bio-Products, Nanjing, 210014 China; 8 Key Laboratory of Animal Diseases Diagnostic and Immunology, Ministry of Agriculture, College of Veterinary Medicine, Nanjing Agricultural University, Nanjing, 210095 China; 9 Yunnan Agricultural University College of Animal Science & Technology, Kunming, Yunnan 650201 China; 10 College of Veterinary Medicine, Auburn University, Auburn, AL USA; 11 Anhui Province Key Laboratory of Veterinary Pathobiology and Disease Control, Anhui Agricultural University, Hefei, 230036 P.R. China

## Abstract

Antimicrobial resistance against colistin has emerged worldwide and is threatening the efficacy of colistin treatment of multi-resistant Gram-negative bacteria. In this study, PCRs were used to detect *mcr* genes (*mcr-1*, *mcr-2*, *mcr-3*) in 213 anal and 1,339 nasal swabs from pigs (n = 1,454) in nine provinces of China, and 1,696 cloacal and 1,647 oropharyngeal samples from poultry (n = 1,836) at live-bird markets in 24 provinces. The *mcr-1* prevalences in pigs (79.2%) and geese (71.7%) were significantly higher than in chickens (31.8%), ducks (34.6%) and pigeons (13.1%). The *mcr-2* prevalence in pigs was 56.3%, significantly higher than in chickens (5.5%), ducks (2.3%), geese (5.5%) and pigeons (0%). The *mcr-3* prevalences in pigs (18.7%), ducks (13.8%) and geese (11.9%) were significantly higher than in chickens (5.2%) and pigeons (5.1%). In total, 173 pigs and three chickens were positive for all three *mcr* genes. The prevalences of the *mcr* were significantly higher in nasal/oropharyngeal swabs than in the anal /cloacal swabs. Phylogenetic studies identified 33 new *mcr-2* variants and 12 new *mcr-3* variants. This study demonstrates high prevalences of *mcr* in pigs and poultry in China, and indicates there is need for more thorough surveillance and control programs to prevent further selection of colistin resistance.

## Introduction

Antimicrobial resistance is recognized as one of the most serious global health threats with the ESKAPE group of pathogens being a large problem^1^. Few treatment options are left and this has triggered the reintroduction of the older and less user-friendly antibiotic colistin^2^. However, the usefulness of colistin as a last resort antimicrobial is now compromised by the presence of an increasing number of mobile colistin resistance genes (*mcr*). To date, five different *mcr* and their variants have been described, mainly *mcr-1* (11 variants)^3–21^, *mcr-2* (three variants)^11–18,21,22^, *mcr-3* (ten variants)^19,20,23–26^, *mcr-4*^27^ and *mcr-5*^28^. The *mcr-1*, *mcr-2* and *mcr-3* were originally described on plasmids in *Enterobacteriaceae* but have recently been identified on the chromosomes of *Moraxella* spp. and *Aeromonas veronii*^21,24,29,30^. *mcr-4* and *mcr-5* have only been described very recently, after we carried out our study in which we used short amplicon PCRs to determine the prevalence of the *mcr-1*, *mcr-2* and *mcr-3* in swabs from the initial and terminal alimentary system of pigs and poultry in China. Positive samples were tested with long amplicon PCRs for the three *mcr* genes that were used for sequencing and comparative studies.

## Results

### PCRs for *mcr*

The detection limit for real-time PCRs with short amplicons was one gene copy in a 20 µL reaction mixture, and the detection limit was 50 copies per reaction for conventional long amplicon PCRs. The established PCRs for *mcr-1*, *mcr-2* and *mcr-3* in this study amplified only the intended *mcr* target and not the other *mcr*^31^.

### Prevalence of *mcr-1*

**Figure 1:**
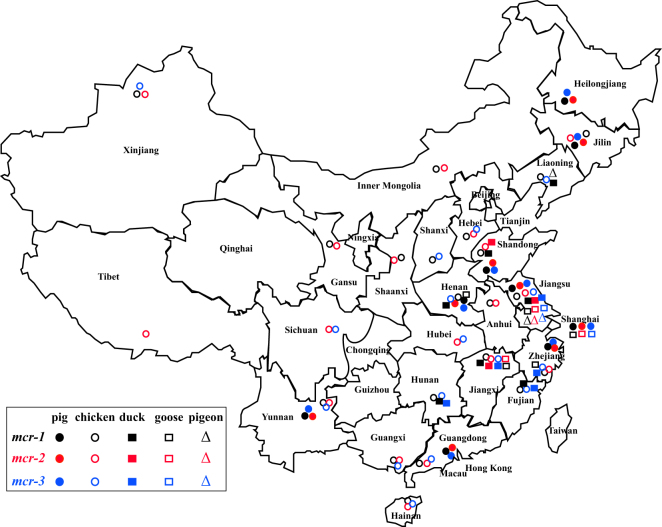
Distribution of *mcr-1*, *mcr-2* and *mcr-3* in pigs and poultry. The *mcr* were detected in specimens from 25 provinces/municipalities of China (*mcr-1*: 2,147/4,895; *mcr-2*: 926/4,895; *mcr-3*: 409/4,895). The prevalences of the *mcr* were much higher in pigs (*mcr-1*: 79.2%, 1,152/1,454; *mcr-2*: 58.4%, 818/1454; *mcr-3*: 17.3%, 272/1454) than in poultry (*mcr-1*: 33.0%, 606/1,836; *mcr-2*: 4.8 88/1,836; *mcr-3*: 5.7%, 105/1,836). The colors (dark, red, blue), shapes (circle, rectangle, triangle) and positions of filled and empty graphics indicate the *mcr* positivity, species sampled and the cities involved, respectively. The Adobe Illustrator CS 11.0.0 was used to create the map in this study.

**Figure 2:**
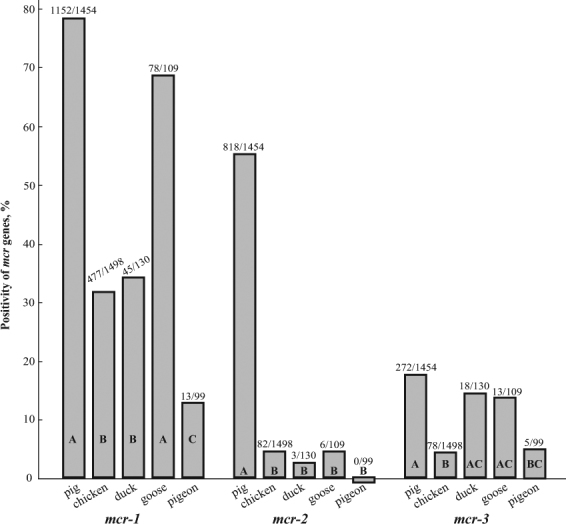
Prevalences of *mcr-1*, *mcr-2* and *mcr-3* in swabs from pigs and poultry. The prevalences of *mcr-1* in chickens (31.8%) and ducks (34.6%) were significantly lower than those in pigs (79.2%) and geese (71.7%), but significantly higher than in pigeons (13.1%). The *mcr-2* prevalence in pigs was 56.3%, significantly higher than in chickens (5.5%), ducks (2.3%), geese (5.5%) and pigeons (0%). The prevalences of the *mcr-3* in pigs (18.7%), ducks (13.8%) and geese (11.9%) were significantly higher than in chickens (5.2%) and pigeons (5.1%). Different letters within bars indicate statistical significance across species, as determined by multiple Pearson Chi-Square test via comparing proportions between two species with Bonferroni adjusted p-values.

The *mcr-1* was identified from pigs sampled in all nine provinces, and poultry in 21 of the 24 provinces of China we studied (Fig. 1). The *mcr-1* specific PCR identified the gene in 83.6% of anal (178/213) and 79.0% of nasal swabs (1,058/1,139) from pigs, and 25.7% of cloacal (436/1,696) and 28.8% of oropharyngeal swabs (475/1,647) from poultry (1,498 chickens; 130 ducks; 109 geese; 99 pigeons) (Table 1, Tables S1–S5). Overall, the *mcr-1* prevalence in chickens and ducks was significantly lower than in pigs and geese, but significantly higher than in pigeons (Fig. 2).

**Table 1:** Prevalences of *mcr-1*, *mcr-2* and *mcr-3* in samples from pigs and poultry in this study.

**Province**	**pig**			**chicken**			**duck**			**goose**			**pigeon**		
	*mcr-1*	*mcr-2*	*mcr-3*	*mcr-1*	*mcr-2*	*mcr-3*	*mcr-1*	*mcr-2*	*mcr-3*	*mcr-1*	*mcr-2*	*mcr-3*	*mcr-1*	*mcr-2*	*mcr-3*
Anhui				61.8% (21/34)	5.9% (2/34)	0% (0/34)									
Fujian				51.4% (18/35)	0% (0/35)	2.9% (1/35)	63.6% (21/33)	0% (0/33)	18.2% (6/33)				0% (0/13)	0% (0/13)	0% (0/13)
Gansu				8.8% (5/57)	1.8% (1/57)	0% (0/57)									
Guangdong	62.5% (25/40)	62.5% (25/40)	17.5% (7/40)	6.2% (4/65)	3.1% (2/65)	7.7% (5/65)	0% (0/4)	0% (0/4)	0% (0/4)						
Guangxi				4.6% (6/130)	10.8% (14/130)	5.4% (7/130)	0% (0/10)	0% (0/10)	0% (0/10)						
Hainan				77.1% (54/70)	1.4% (1/70)	4.3% (3/70)									
Hebei				2.1% (2/96)	8.3% (8/96)	1.0% (1/96)	0% (0/6)	0% (0/6)	0% (0/6)				0% (0/34)	0% (0/34)	0% (0/34)
Heilongjiang	95% (57/60)	96.7% (58/60)	16.7% (10/60)												
Henan	63.5% (40/63)	61.9% (39/63)	36.5% (23/63)	21.4% (12/56)	0% (0/56)	1.8% (1/56)	71.4% (5/7)	0% (0/7)	0% (0/7)	85.7% (6/7)	0% (0/7)	0% (0/7)			
Hubei				0% (0/64)	12.5% (8/64)	3.1% (2/64)	0% (0/6)	0% (0/6)	0% (0/6)						
Hunan				4.3% (3/70)	0% (0/70)	4.3% (3/70)									
Inner Mongolia				1.5% (1/65)	1.5% (1/65)	0% (0/65)				0% (0/5)	0% (0/5)	0% (0/5)			
Jiangsu	71.9% (424/590)	48.8% (288/590)	8.6% (51/590)	77.3% (119/154)	0.6% (1/154)	23.4% (36/154)	29.0% (9/31)	0% (0/31)	25.8% (8/31)	88.9% (8/9)	0% (0/9)	100% (9/9)	23.9% (11/46)	0% (0/46)	10.9% (5/46)
Jiangxi				66.7% (46/49)	40.8% (20/49)	12.2% (6/49)	54.5% (6/11)	18.2% (2/11)	27.3% (3/11)	88.9% (8/9)	44.4% (4/9)	0% (0/9)			
Jilin	100% (52/52)	50.8% (32/63)	25.4% (16/63)	27.1% (19/70)	2.9% (2/70)	0% (0/70)									
Liaoning				64.9% (24/37)	0% (0/37)	5.4% (2/37)	28.6% (2/7)	0% (0/7)	0% (0/7)				33.3% (2/6)	0% (0/6)	0% (0/6)
Shaanxi				11.4% (8/70)	1.4% (1/70)	0% (0/70)									
Shandong	41.7% (25/60)	11.7% (7/60)	26.7% (16/60)	6.8% (4/59)	1.7% (1/59)	0% (0/59)	66.7% (2/3)	33.3% (1/3)	0% (0/3)	0% (0/8)	0% (0/8)	0% (0/8)			
Shanghai	47.2% (25/53)	22.6% (12/53)	3.8% (2/53)							78.6% (55/70)	2.9% (2/70)	5.7% (4/70)			
Shanxi				60% (12/20)	0% (0/20)	5.0% (1/20)									
Sichuan				0% (0/70)	8.6% (6/70)	1.4% (1/70)									
Tibet				0% (0/31)	3.3% (1/30)	0% (0/30)									
Xinjiang				82.9% (58/70)	2.9% (2/70)	4.3% (3/70)									
Yunnan	100% (130/130)	38.5% (50/130)	41.5% (54/130)	85.7% (60/70)	12.9% (9/70)	7.1% (5/70)									
Zhejiang	92.4 (365/395)	77.7% (307/395)	23.5% (93/395)	1.8% (1/57)	3.5% (2/57)	1.8% (1/57)	0% (0/12)	0% (0/12)	8.3% (1/12)	100% (1/1)	0% (0/1)	0% (0/1)			
**Total**	79.2% (1152/1454)	56.3% (818/1454)	18.7% (272/1454)	31.8% (477/1498)	5.5% (82/1498)	5.2% (78/1498)	34.6% (45/130)	2.3% (3/130)	13.8% (18/130)	71.6% (78/109)	5.5% (6/109)	11.9% (13/109)	13.1% (13/99)	0% (0/99)	5.1% (5/99)

Geese had the highest *mcr-1* prevalence (65.1%, 71/109 of cloacal swabs; 45.0%, 49/109 of oropharyngeal swabs), followed by chickens (23.4%, 323/1,383 of cloacal swabs; 28.6%, 386/1,350 of oropharyngeal swabs), ducks (24.6%, 30/122 of cloacal swabs; 21.3%, 26/122 of oropharyngeal swabs) and pigeons (14.6%, 12/82 of cloacal swabs; 21.2%, 14/66 of oropharyngeal swabs).

### Prevalence of *mcr-2*

**Figure 3:**
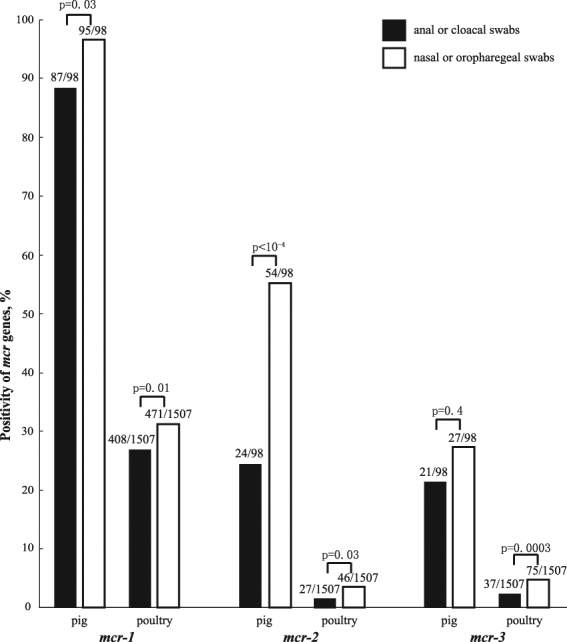
Prevalence of *mcr-1*, *mcr-2* and *mcr-3* in the initial and terminal alimentary system of pigs and poultry. Swabs from both initial and terminal alimentary systems were collected from 98 pigs and 1,507 poultry in this study. For all three *mcr* genes in swabs from pigs and *mcr-1* and *mcr-2* in swabs from poultry, the prevalences in nasal/oropharyngeal were significantly higher than in the anal /cloacal swabs.

The *mcr-2* was identified in pigs sampled from all nine provinces, and poultry in 19 of the 24 provinces of China we studied (Fig. 1). The prevalence of *mcr-2* in pigs was 56.3% (nasal: 58.4%, 782/1,339; anal: 23.0%, 49/213), being significantly higher than in poultry (oropharyngeal: 3.6%, 60/1,647; cloacal: 2.1%, 35/1,696) (Table 1, Fig. 3, Tables S1–S5). The *mcr-2* gene was identified in chickens (5.5%, 82/1,498), ducks (2.3%, 3/130) and geese (5.5%, 6/109), but not in pigeons (0/99).

### Prevalence of *mcr-3*

Overall, the *mcr-3* was detected by PCR in 8.4% of samples (409/4,895) including anal (23%, 49/213) and nasal swabs (17.3%, 232/1,339) from pigs, and oropharyngeal (4.9%, 81/1,647) and cloacal swabs (2.8%, 47/1,696) from poultry (Table 1, Fig. 3, Tables S1–S5). Positive *mcr-3* PCRs were obtained from pigs in all nine provinces, and poultry sampled in 17 of the 24 provinces (chicken: 5.2%, 78/1,498; duck: 13.8%, 18/130; goose: 11.9%, 13/109; pigeons: 5.1%, 5/99) (Figs 1, 2).

### Co-occurrence of *mcr-1*, *mcr-2* and *mcr-3*

One-hundred and seventy three PCRs on anal and nasal swabs from 1,454 pigs were positive for all three colistin resistance genes (Table 2). Dual positivity was identified in 730 pigs for *mcr-1* and *mcr-2*, 267 pigs for *mcr-1* and *mcr-3*, and 177 pigs for *mcr-2* and *mcr-3*.

**Table 2:** Co-occurrence of *mcr* genes in swabs from pigs and poultry in this study.

**Positive for** ***mcr*** **genes**	**pig**			**chicken**			**duck**			**goose**			**pigeon**		
	Nasal (1339)	Anal (213)	Total* (1454)	Oral (1350)	Cloacal (1383)	Total (1498)	Oral (122)	Cloacal (122)	Total (130)	Oral (109)	Cloacal (109)	Total (109)	Oral (82)	Cloacal (66)	Total (99)
*mcr-1*	79.0% (1058/1339)	83.6% (178/213)	79.2% (1152/1454)	28.6% (386/1350)	23.4% (323/1383)	31.8% (477/1498)	21.3% (26/122)	24.6% (30/122)	34.6% (45/130)	45.0% (49/109)	65.1% (71/109)	71.6% (78/109)	17.1% (14/82)	18.2% (12/66)	13.1% (13/99)
*mcr-2*	58.4% (782/1339)	23.0% (49/213)	56.3% (818/1454)	4.0% (54/1350)	2.2% (31/1383)	5.5% (82/1498)	1.6% (2/122)	0.8% (1/122)	2.3% (3/130)	3.7% (4/109)	2.8% (3/109)	5.5% (6/109)	0% (0/82)	0% (0/66)	0% (0/99)
*mcr-3*	17.3% (232/1339)	23.0% (49/213)	18.7% (272/1454)	3.8% (51/1350)	2.6% (36/1383)	5.2% (78/1498)	13.1% (16/122)	4.1% (5/122)	13.8% (18/130)	10.1% (11/109)	3.7% (4/109)	11.9% (13/109)	3.7% (3/82)	3.0% (2/66)	5.1% (5/99)
*mcr-1* and *mcr-2*	52.1% (698/1339)	20.2% (43/213)	50.2% (730/1454)	1.8% (24/1350)	0.6% (8/1383)	2.1% (31/1498)	0.8% (1/122)	0.8% (1/122)	1.5% (2/130)	2.8% (3/109)	1.8% (2/109)	3.7% (4/109)	0% (0/82)	0% (0/66)	0% (0/99)
*mcr-1* and *mcr-3*	17.0% (227/1339)	23.0% (49/213)	18.4% (267/1454)	2.2% (30/1350)	1.9% (26/1383)	3.2% (48/1498)	4.1% (5/122)	2.5% (3/122)	5.4% (7/130)	6.4% (7/109)	2.8% (3/109)	8.3% (9/109)	2.4% (2/82)	0% (0/66)	2.0% (2/99)
*mcr-2* and *mcr-3*	12.3% (165/1339)	6.1% (13/213)	12.2% (177/1454)	0.2% (3/1350)	0.1% (1/1383)	0.3% (4/1498)	0% (0/122)	0% (0/122)	0% (0/130)	0% (0/109)	0% (0/109)	0% (0/109)	0% (0/82)	0% (0/66)	0% (0/99)
*mcr-1*, *mcr-2*, and *mcr-3*	12.0% (161/1339)	6.1% (13/213)	11.9% (173/1454)	0.2% (3/1350)	0% (0/1383)	0.2% (3/1498)	0% (0/122)	0% (0/122)	0% (0/130)	0% (0/109)	0% (0/109)	0% (0/109)	0% (0/82)	0% (0/66)	0% (0/99)

^*^Total means the total number of the assayed animals. Under the column of Total, when one of the Nasal/Oral and Anal/cloacal swabs was positive, this animal was considered to be positive.

Three chickens were positive for *mcr-1*, *mcr-2* and *mcr-3*. Both of the *mcr-1* and *mcr-2* were identified in 37 birds (31 chickens; 2 ducks; 4 geese) while 66 birds (48 chickens, 7 ducks, 9 gooses, 2 pigeons) were positive for *mcr-1* and *mcr-3* and 4 chickens were positive for both *mcr-2* and *mcr-3* (Table 2).

### Comparison of *mcr* between samples from initial and terminal alimentary systems

Swabs from both of the initial and terminal alimentary systems were collected from 98 pigs and 1,507 poultry. In general, the *mcr* prevalences in nasal/oropharyngeal swabs were significantly higher than in anal/cloacal swabs (Fig. 3).

For the 98 pigs with both anal and nasal swabs available, 84 were *mcr-1* positive in both samples, three had only positive anal swabs, and 11 only had positive nasal swabs. For the *mcr-2*, only 13 pigs were positive in both swabs, 11 were positive for anal swabs only and 41 for cloacal swabs only. Nine pigs were positive for *mcr-3* in both swabs with 12 having only nasal swabs positive and 18 having only anal swabs positive (Fig. 3, Tables 1, 2, Table S1).

Both oropharyngeal and cloacal swabs were positive for *mcr-1* in 285 poultry (230 chickens, 11 ducks, 42 geese, 2 pigeons), for *mcr-2* in four poultry (3 chickens, 1 goose) and for *mcr-3* in 14 poultry (9 chickens, 3 ducks, 2 geese). For the *mcr-1*, only the cloacal swab was positive for 123 birds (75 chickens, 19 ducks, 29 geese) and only the oropharyngeal swab was positive for 186 birds (152 chickens, 15 ducks, 7 geese, 12 pigeons). For the *mcr-2*, 23 birds only had positive cloacal swabs (20 chickens, 1 duck, 2 geese) while 42 only had positive oropharyngeal swabs (37 chickens, 2 ducks, 3 geese). Cloacal swabs of 23 birds (18 chickens, 2 ducks, 2 geese, 1 pigeon) were positive for *mcr-3*, while oropharyngeal swabs of 61 birds (36 chickens, 13 ducks, 9 geese, 3 pigeons) were positive for *mcr-3* (Fig. 3, Table 1, Tables 2, S2–S5).

### Phylogenetic comparison

We successfully sequenced long amplicons (1,497-bp) of 66 *mcr-1* PCRs (31 pigs, 23 chickens, 6 ducks, 3 geese, 3 pigeons), 33 *mcr-2* PCRs (28 pigs, 4 chickens, 1 duck) and 25 *mcr-3* PCRs (14 pigs, 2 chickens, 2 ducks, 7 geese). The nucleotide sequences of the *mcr-1* amplified in this study were identical to those of the *mcr*-1 previously reported in bacteria from flies (MF598564)^31^ and *Escherichia coli* from pigs in China (KP347127)^3^.

**Figure 4:**
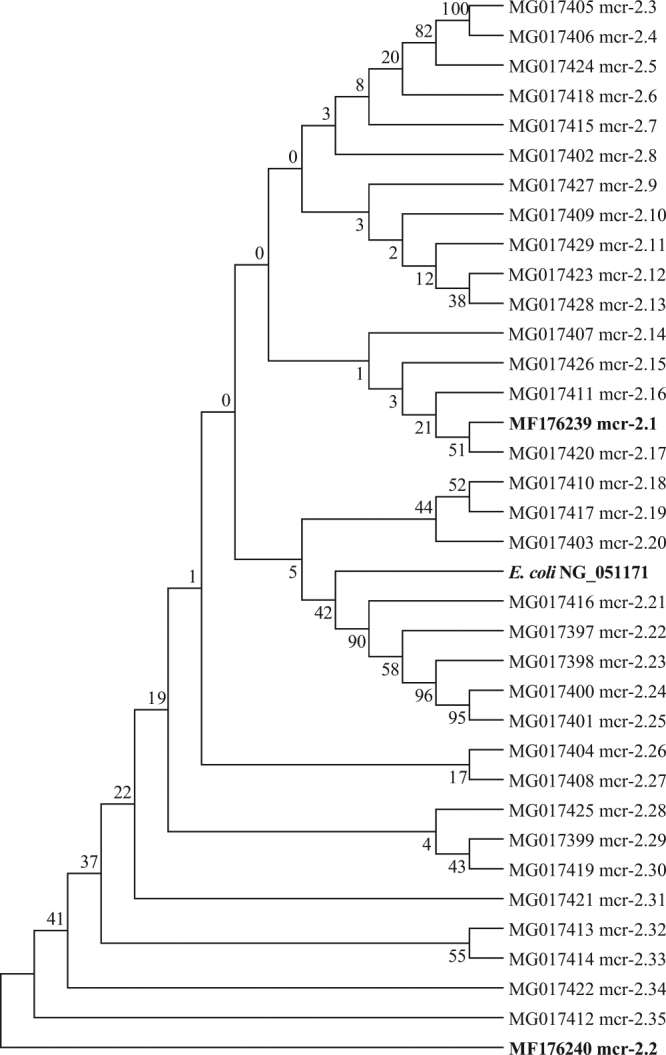
Phylogenetic analysis of colistin resistance gene *mcr-2*. The nucleotide sequences of *mcr-2* gene (1,042-bp) identified in this study are compared with representative sequences from NCBI (in bold font) using the Neighbor-Joining method. The optimal tree with the sum of branch length (0.419) is shown. The percentage of replicate trees in which the associated taxa clustered together in the bootstrap test (500 replicates) are shown next to the branches. The evolutionary distances were computed using the Kimura 2-parameter method and are in the units of the number of base substitutions per site. All positions containing gaps and missing data were eliminated. Evolutionary analyses were conducted in MEGA6.

The nucleotide sequences obtained for the *mcr-2* revealed 33 new variants of *mcr-2* (mcr-2.3 to mcr-2.35) which had high levels of similarity (95.9–99.9%). They also had high similarity (95.8% and 98%) with the *mcr-2* from *E*. *coli* KP37 isolated from pigs and cattle in Belgium (NG_051171). The other two variants we found had lower similarity: variant mcr-2.1 (MF176239) with 95.4% to 97.5% and variant mcr-2.2 (MF176240) with 87.0% to 88.4% (Fig. 4). The deduced amino acid sequences (347-aa) of the mcr-2.3 to mcr-2.35 were 98.6%-100% identical to those of the *E*. *coli* KP37 strain from Belgium (Figure S1).

**Figure 5:**
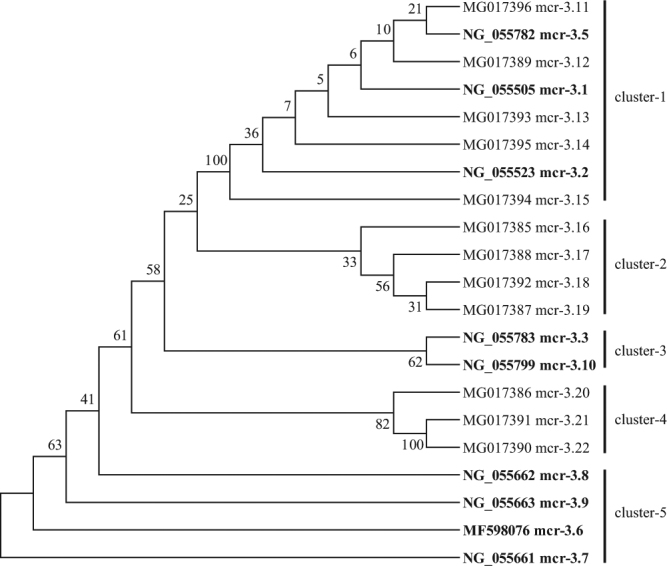
Evolutionary relationships of *mcr-3* sequences obtained in this study. The evolutionary history of *mcr-3* nucleotides (1,064-bp) identified in this study and NCBI (in bold font) was inferred using the Neighbor-Joining method. The optimal tree with the sum of branch length (0.0372) is shown. The percentage of replicate trees in which the associated taxa clustered together in the bootstrap test (500 replicates) are shown next to the branches. The evolutionary distances were computed using the Kimura 2-parameter method and are in the units of the number of base substitutions per site. After eliminating the positions containing gaps and missing data, there were a total of 1,063 positions in the final dataset. Evolutionary analyses were conducted in MEGA6.

We also identified 12 new variants of the *mcr-3* (mcr-3.11 to mcr-3.22) which formed three potential clusters with phylogenetic tree analysis (Fig. 5). The variants in the first cluster (cluster 1) had 0–2 nucleotides mismatches and comprised five from this study (mcr-3.11 to mcr-3.15) and three reported before (mcr-3.1, NG_055505; mcr-3.2, NG_055523, mcr-3.5 (NG_055782). Those in cluster 2 had 2–21 nucleotide mismatches and consisted of seven variants from this study (mcr-3.16 to mcr-3.22) and two described before (mcr-3.3, NG_055783; mcr-3.10, NG_055799). The remaining four variants (mcr-3.6, MF598076; mcr-3.7, NG_055661; mcr-3.8, NG_055662; mcr-3.9, NG_055663) were polymorphic with the other *mcr-3* (Fig. 5). Not all sequence mutations lead to differences at the amino acid level with the variants in cluster 1 having 0–2 different amino acids (Figure S2) and those in cluster 2 having 0–8 amino acid changes (Figure S2).

All the nucleotide sequences were submitted to GenBank with accession numbers MG017397 to MG017429 for *mcr-2*, and MG017385 to MG017396 for *mcr-3*.

## Discussion

While colistin is a last-line antibiotic used to treat multidrug resistant Gram-negative bacteria, its efficacy is being compromised by the recently detected mobile colistin resistance genes, *mcr-1*^3^, and subsequently *mcr-2*, *mcr-3*, *mcr-4*, *mcr-*5^11,24,27,28^. Our study has shown that the *mcr-1*, *mcr-2* and *mcr-3* occurred widely in pigs and poultry from China (Table 1, Tables S1–S5). The prevalences we found were considerably higher than those previously reported in China and elsewhere in the world^3–26,29,30^ which were based on the presence of the *mcr* in bacterial isolates. Bacterial isolation, however, is cumbersome, costly, and time-consuming, and it is also selective in that samples must be appropriately collected, transported and stored to maximize the number of viable bacteria with the *mcr* that can be selectively cultured. Directly testing samples with the sensitive and specific *mcr*-PCRs we developed meant that we avoided the limitations of isolation and the resultant underestimation of the *mcr* genes that the technique involves. The phenomenon whereby direct sample testing gives far higher prevalences than testing of isolates from samples has been termed the ‘phantom resistome’^32^ and indicates that the existing data on the prevalence of the *mcr-1*, *mcr-2* and *mcr-3* in China greatly underestimates the real situation. We note, however, that both methods are needed to understand the epidemiology of resistance. While direct PCR testing is ideal for the rapid estimation of risk and risk analysis, it does not readily enable investigations of movement of resistance between bacteria of the same or different species. As we did not base the study on the isolation of the bacteria, we are not sure which bacteria carry the resistance genes we identified, or the mobile genetic elements on which the genes are carried. Previous studies have shown the *mcr-1*, *mcr-2* and *mcr-3* can be on plasmids in *Enterobacteriaceae* and chromosomes in *Moraxella* spp.^21,29,30^.

The high levels of the *mcr-1* we found in the pigs and the poultry we studied from 21 of the 24 provinces in China (Table 1, Tables S1–S5) is consistent with previous reports from China^20,31–33^ and is likely associated with the prolonged and widespread use of colistin as a growth promoter in food animals.

We also found a high prevalence of the *mcr-2* in both pigs and poultry which is surprising as this gene has only been found in a limited number of other studies^11,12,14,15,17,18,22,27^. This is likely because our PCR for *mcr* is more sensitive in detecting the resistance gene than the conventional methods relying on bacterial isolation as discussed above. The PCR, however, did not enable us to determine the prevalences of *mcr-2* in the different bacterial populations in our samples and it is possible that only a small part of the bacterial population carries this resistance gene. In studies on selectively isolated bacteria however, its presence may be masked if only low numbers of *mcr*-2 positive strains are present and higher numbers of strains with *mcr-1* or mutational mediated resistance strains.

The *mcr-3* gene was first characterized on a IncHI2-type plasmid pWJ1 from porcine *E*. *coli* in China^23^ and later shown to be present in bacteria isolated from humans from Denmark, chickens from China, cattle from Spain^19,20,24–26^, and flies in China^31^. Our findings indicate that the gene is quite widespread, as we found it in samples from pigs and poultry from many provinces in China, albeit at low prevalence in some cases. Further studies in other countries and from distinct sources should be performed to determine how this gene is spread and its overall role in colistin resistance.

The *mcr-1* sequences were highly conserved in the pigs and various poultry we studied and identical to sequences described before^3^. However, the nucleotide sequences of the *mcr-2* we found varied and we identified 33 variants which were very similar (21–44 nucleotide mismatches) to those originally reported by Xavier BB *et al*.^22^. Although highly polymorphic at the nucleotide level, there were only few differences at the amino acid level (0–5 mismatches). It remains unclear whether these mutations influence the level of colistin resistance. Furthermore, these data are difficult to interpret, as we do not know in which bacterial species the gene resides. Differences in codon usage of the different bacterial species might have been the cause of the high variability found here. Further studies are warranted to explore the influence of *mcr-2* mutation on colistin resistance.

Alignment of *mcr-3* nucleotide sequences obtained in this study (mcr-3.11 to mcr-3.22) and those reported before (mcr-3.1 to mcr-3.10) revealed five clusters. Those in cluster 1 were obtained from pigs and people, except for the mcr-3.12 which was obtained from a goose in our study. All the sequences in clusters 2, 3 and 4 were derived from poultry, while sequences of cluster 5 were from fish and turkeys. The host specificity of the *mcr-3* was demonstrated at sites 188, 286, 302, 321, 326, 330 and 347 of the amino acid sequences. Further studies are needed to investigate the possible interrelationship between the host-specificity of nucleotide and amino acid sequences and the epitopes of colistin resistance.

Our comparison of the prevalence of the *mcr* genes in different ecosystems on the same animal, that is both nasal/oropharyngeal swabs and anal/cloacal swabs from pigs and chickens, respectively, showed that most positive animals had *mcr* containing bacteria at both sites. In the remaining animals, more were only *mcr* positive in nasal/ oropharyngeal swabs than only positive in anal/cloacal swabs. These results are consistent with bacteria in these ecosystems being exposed to colistin during ingestion of food containing the additive, and also during digestion of this food. It should also be noted, however, that there can be substantial concentration of colistin in dust^34^ and this might also be a source of respiratory mucosa exposure. Whereas feces are most commonly considered as the major factor in the transfer and spread of the *mcr* genes, our data showing significantly higher *mcr* prevalences in nasal/oropharyngeal swabs than in anal/cloacal swabs suggests that both saliva and respiratory secretions might also play important roles. The identification of *mcr* variants in *Moraxella* species (*mcr-1* and *mcr-2*)^21,29,30^ and *Aeromonas veronii* (*mcr-3*)^24^ indicates there are organisms outside the *Enterobacteriaceae* that contribute to colistin resistance and these might be responsible for the differences in prevalences that we found.

In summary, our study indicates that the *mcr-1*, *mcr-2* and *mcr-3* are relatively common and widespread in food producing animals of China. The high sequence variability of some of the genes indicates there is ongoing evolution, probably in different bacterial species. Future studies should focus on the bacterial species carrying these genes and the localization of these genes in the microorganisms.

## Materials and Methods

### Ethics statement

This study was reviewed and approved by the Institutional Animal Care and Use Committee of Yangzhou University College of Veterinary Medicine, and was performed in accordance with the relevant guidelines and regulations.

### Swab samples from pigs and poultry

In 2016, 232 anal and 1,339 nasal swabs from apparently healthy pigs (n = 1,552) in nine provinces were collected. In addition, 1,690 cloacal and 1,628 oropharyngeal samples from poultry (n = 1,836) at 38 live-bird markets in 24 different provinces in China were collected in 2014 (Fig. 1)^35^. Both of the swabs from the initial and terminal alimentary systems were collected from 98 pigs and 1,507 poultry in this study.

To collect an oropharyngeal swab sample from poultry, the swab was introduced into the bird’s mouth and rubbed around the tracheal opening and up along the choanae. To collect the cloacal swabs from poultry, the swab was gently introduced into the cloaca through the vent and gently twirled at an appropriate depth to ensure contact with the mucous membranes.

To collect the porcine nasal swabs, the nose was wiped with a dry piece of paper and the swab inserted into the ventral nasal passage and rotated through ninety degrees for three seconds. To collect anal swabs from pigs, the swab was inserted one cm into the rectum while being rotated.

Following sampling, the swabs were immersed in 400 μl DNA/RNA Stabilization Buffer (Roche Molecular Biochemicals, IN, USA) in sterile tubes and frozen at −80 °C until DNA was extracted.

### DNA extraction

Swabs were centrifuged in the DNA/RNA Stabilization Buffer (3,000×g, 4 °C for 5 min) and DNA was extracted from the supernatants using either the High Pure PCR Template Preparation Kit (Roche Diagnostic, USA) following the manufacturer’s instructions for the oropharyngeal and nasal swabs or the QIAamp DNA Stool Mini Kit (Qiagen, USA) for the cloacal and anal swabs^31^.

### PCR assays

In this study, we used previously described PCRs^31^. These PCRs do not amplify the chromosomal *mcr* genes described in *Moraxella hydrophila* and *Aeromonas veronii*. The PCRs for the *mcr-1*, *mcr-2* and *mcr-*3 genes were performed on a Roche Light-Cycler 480II PCR instrument^31^. The short amplicon PCRs for *mcr-1*(342-bp), *mcr-2* (282-bp) and *mcr-3* (267-bp) were used to establish prevalence data and positive samples were tested with the long amplicon PCRs for *mcr-1* (1,497-bp), *mcr-2* (1,042-bp) and *mcr-3* (1,063-bp). Positive long amplicon PCR products were verified by gel electrophoresis and sequenced using upstream and downstream primers (BGI, Shanghai, China). Sequences obtained were compared with those published in the NCBI database (www.ncbi.nlm.nih.gov) using the Clustal Multiple Alignment Algorithm. Negative controls consisting of sterile molecular grade water were used to detect cross-contamination during DNA purification and PCR processing.

### Phylogenetic analysis

The sequences from this study and those from GenBank for the *mcr-1*, *mcr-2* and *mcr-3* were aligned using the MEGA 6.0 software. Based on these alignments, phylogenetic trees were constructed by the neighbor-joining method using the Kimura 2-parameter model with MEGA 6.0. Bootstrap values were calculated using 500 replicates.

### Statistical analysis

Multiple Pearson Chi-Square test was used for comparing differences between animal species as well as between anal/oropharyngeal and nasal/oral swabs with Bonferroni adjusted p-values.

## Electronic supplementary material

Supplementary Information 
