# Supplementary material for: Molecular detection of colistin resistance genes (mcr-1, mcr-2 and mcr-3) in nasal/oropharyngeal and anal/cloacal swabs from pigs and poultry
Source: Sci Rep. 2018 Feb 27;8:3705. doi: 10.1038/s41598-018-22084-4 (PMC5829079; doi:10.1038/s41598-018-22084-4)
Supplement: Supplementary file 1 — Supplementary Information [file 41598_2018_22084_MOESM1_ESM.pdf]

Molecular detection of colistin resistance genes (*mcr-1*, *mcr-2* and *mcr-3*) in nasal/oropharyngeal and anal/cloacal swabs from pigs and poultry

Jilei Zhang[1]*, Li Chen[1]*, Jiawei Wang[1], Afrah Kamal Yassin[1,2], Patrick Butaye[3, 4], Patrick Kelly[3], Jiansen Gong[5], Weina Guo[6], Jing Li[1], Min Li[1], Feng Yang[1], Zhixing Feng[7], Ping Jiang[8], Chunlian Song[9], Yaoyao Wang[1], Jinfeng You[1], Yi Yang[1], Stuart Price[10], Kezong Qi[11], Yuan Kang[10], Chengming Wang[1, 10]**

[1] Jiangsu Co-Innovation Center for Prevention and Control of Important Animal Infectious Diseases and Zoonoses, Yangzhou University College of Veterinary Medicine, Yangzhou, Jiangsu, 225009, PR China

[2] Department of Food Hygiene and safety, Faculty of Public and Environmental Health, Khartoum University, Sudan

[3] Department of Biosciences, Ross University School of Veterinary Medicine, P.O. Box 334, Basseterre, St. Kitts, West Indies

[4] Department of Pathology, Bacteriology and Poultry diseases, Faculty of Veterinary Medicine, Ghent University, Ghent, Belgium

[5] Poultry Institute, Chinese Academy of Agricultural Sciences, Yangzhou, Jiangsu, China

[6] College of Animal Science, Anhui Science and Technology University, Bengbu, 230001, China

[7] Institute of Veterinary Medicine, Jiangsu Academy of Agricultural Sciences, Key Laboratory of Veterinary Biological Engineering and Technology, Ministry of Agriculture; National Center for Engineering Research of Veterinary Bio-Products, Nanjing 210014, China

[8] Key Laboratory of Animal Diseases Diagnostic and Immunology, Ministry of Agriculture, College of Veterinary Medicine, Nanjing Agricultural University, Nanjing 210095, China

[9] Yunnan Agricultural University College of Animal Science & Technology, Kunming, Yunnan, 650201, China

[10] College of Veterinary Medicine, Auburn University, Auburn, AL, USA

[11] Anhui Province Key Laboratory of Veterinary Pathobiology and Disease Control, Anhui Agricultural University, Hefei 230036, PR China

* The authors contributed equally to this work.

** Correspondence: Chengming Wang, College of Veterinary Medicine, Auburn University, Auburn, AL, USA; Tel: +1-334-844-2601; Fax: +1-334-844-2152; Email: wangche@auburn.edu

Table S1. Prevalences of *mcr* genes in anal and nasal swabs in pigs.

Province	City	Positive /total samples		
		mcr-1	***mcr-2***	***mcr-3***
Jiangsu	Yangzhou	A: 27/51	A: 12/51	A: 2/51
Yunnan	Kunming	A: 64/64<br>N: 66/66	A: 13/64<br>N: 37/66	A: 26/64<br>N: 28/66
Zhejiang	Ningbo	A: 48/48<br>N: 50/50<br>T*: 50/50	A: 8/48<br>N: 18/50<br>T: 21/50	A: 17/48<br>N: 19/50<br>T: 28/50
	Shaoxing	A: 39/50<br>N: 312/345<br>T: 315/345	A: 16/50<br>N: 278/345<br>T:286/345	A: 4/50<br>N: 62/345<br>T:65/345
Guangdong	Jiangmen	N: 25/40	N: 25/40	N: 7/40
Heilongjiang	Haerbin	N: 57/60	N: 58/60	N: 10/60
Henan	Xihua	N: 40/63	N: 39/63	N: 23/63
Jiangsu	Dafeng	N: 21/37	N: 24/37	N: 5/37
	Jiangyan	N: 4/31	N: 6/31	N: 0/31
	Liyang	N: 10/23	N: 12/23	N: 0/23
	Nanjing	N: 317/394	N: 190/394	N: 37/394
	Taixing	N: 18/18	N: 16/18	N: 6/18
	Wuxi	N: 27/36	N: 28/36	N: 1/36
Jilin	Changchun	N: 61/63	N: 32/63	N: 16/63
Shandong	Jining	N: 25/60	N: 7/60	N: 16/60
Shanghai	Shanghai	N: 25/53	N: 12/53	N: 2/53

*T: total number of assayed animals.

Table S2. Prevalences of *mcr* genes in cloacal (C) and oropharyngeal (O) swabs in chicken.

Province	City	Positive /total samples		
		mcr-1	***mcr-2***	***mcr-3***
Anhui	Fuyang	C: 7/34;<br>O: 20/34;<br>T*: 21/34	C: 1/34;<br>O: 1/34;<br>T: 2/34	T: 0/34
Fujian	Nanping	C: 10/35;<br>O: 12/35;<br>T: 18/35	T: 0/35	C:0/35;<br>O: 1/35;<br>T: 1/35
Gansu	Jingyuan	C: 2/57;<br>O: 3/57;<br>T: 5/57	C:0/57;<br>O: 1/57;<br>T: 1/57	T: 0/57
Guangdong	Zhanjiang	C: 2/65;<br>O: 2/54;<br>T: 4/65	C: 0/65;<br>O: 2/54;<br>T: 2/65	C: 1/65;<br>O: 4/54;<br>T: 5/65
Guangxi	Beihai	C: 4/65;<br>O: 2/65;<br>T: 6/130	C: 5/65;<br>O: 9/65;<br>T: 14/130	C: 1/65;<br>O: 6/65;<br>T: 7/130
Hainan	Wenchang	C: 29/70;<br>O: 53/70;<br>T: 54/70	C: 0/70;<br>O: 1/70;<br>T: 1/70	C: 2/70;<br>O: 2/70;<br>T: 3/70
Hebei	Shijiazhuang	C: 0/46;<br>O: 2/50;<br>T: 2/96	C: 3/46;<br>O: 5/50;<br>T: 8/96	C: 1/46;<br>O: 0%,<br>T: 1/96
Henan	Anyang	C: 8/56;<br>O: 11/56;<br>T: 12/56	T:0/56	C: 1/56;<br>O: 0/56;<br>T: 1/56
Hubei	Wuhan	T: 0/64	C: 3/64;<br>O: 5/53;<br>T: 8/64	C: 1/64;<br>O: 1/53;<br>T: 2/64
Hunan	Yongzhou	C: 1/70;<br>O: 2/70;<br>T: 3/70	T: 0/70	C: 1/70;<br>O: 2/70;<br>T: 3/70
Inner Mongolia	Ulanqab	C: 0/65;<br>O: 1/65;<br>T: 1/65	C: 1/65;<br>O: 0/65;<br>T: 1/65	T: 0/65
Jiangsu	Yangzhou	C: 113/126;<br>O: 92/111;<br>T: 118/126	C: 1/126;<br>O: 0/111;<br>T: 1/126	C: 23/126;<br>O: 21/111;<br>T: 36/126
	Yixing	C: 0/28;<br>O: 1/28;<br>T: 1/28	T: 0/28	T: 0/28

Jiangxi	Xingan	C: 35/49;<br>O: 38/49;<br>T: 46/49	C: 6/49;<br>O: 17/49;<br>T: 20/49	C: 1/49;<br>O: 5/49;<br>T: 6/49
Jilin	Changchun	C: 4/70;<br>O: 17/70;<br>T: 19/70	C: 0/70;<br>O: 2/70;<br>T: 2/70	T: 0/70
Liaoning	Jinzhou	C:14/37;<br>O: 15/37;<br>T: 24/37	T: 0/37	C: 0/37;<br>O: 2/37;<br>T: 2/37
Shaanxi	Yanan	C: 4/70;<br>O: 6/70;<br>T: 8/70	C: 1/70;<br>O: 0/70;<br>T: 1/70	T: 0/70
Shandong	Liaocheng	C: 2/59;<br>O: 2/59;<br>T: 4/59	C: 1/59;<br>O: 0/59;<br>T: 1/59	T: 0/59
Shanxi	Changzhi	C: 9/20;<br>O: 5/20;<br>T: 12/20	T: 0/20	C: 0/20;<br>O: 1/20;<br>T: 1/20
Sichuan	Chengdu	T: 0/70	C: 5/70;<br>O: 1/70;<br>T: 6/70	C: 1/70;<br>O: 0/70;<br>T: 1/70
Tibet	Shigatse	T: 0/30	C: 0/30;<br>O: 1/30;<br>T: 1/30	T: 0/30
Xinjiang	Hoboksar	C: 39/70;<br>O: 48/70;<br>T: 58/70	C: 0/70;<br>O: 2/70;<br>T: 2/70	C: 3/70;<br>O: 0/70;<br>T: 3/70
Yunnan	Yiwei	C: 40/70;<br>O: 53/70;<br>T: 60/70	C: 3/70;<br>O: 6/70;<br>T: 9/70	C: 0/70;<br>O: 5/70;<br>T: 5/70
Zhejiang	Wenzhou	C: 0/57;<br>O: 1/57;<br>T: 1/57	C: 1/57;<br>O: 1/57;<br>T: 2/57	C: 0/57;<br>O: 1/57;<br>T: 1/57

*T: total number of assayed animals.

Table S3. Prevalences of *mcr* genes in cloacal (C) and oropharyngeal (O) swabs in ducks.

Province	City	Positive /total samples		
		mcr-1	***mcr-2***	***mcr-3***
Fujian	Nanping	C: 11/33;<br>O:14/33;<br>T*: 21/33	T: 0/33	C: 2/33;*<br>O: 5/33;<br>T: 6/33
Guangdong	Zhanjiang	T: 0/4	T: 0/4	T: 0/4
Guangxi	Beihai	T: 0/10	T: 0/10	T: 0/10
Hebei	Shijiazhuang	T: 0/6	T: 0/6	T: 0/6
Henan	Anyang	C: 5/7;<br>O: 2/7;<br>T: 5/7	T: 0/7	T: 0/7
Hubei	Wuhan	T: 0/6	T: 0/6	T: 0/6
Jiangsu	Yangzhou	C: 8/10;<br>O: 1/10;<br>T: 8/10	T: 0/10	C: 3/10;<br>O: 7/10;<br>T: 8/10
	Yixing	C: 0/21;<br>O: 1/21;<br>T: 1/21	T: 0/21	T: 0/21
Jiangxi	Xingan	C: 4/11;<br>O: 6/11;<br>T: 6/11	C: 1/11;<br>O: 1/11;<br>T: 2/11	C: 0/11;<br>O: 3/11;<br>T: 3/11
Liaoning	Jinzhou	C: 0/7;<br>O: 2/7;<br>T: 2/7	T: 0/7	T: 0/7
Shandong	Liaocheng	C: 2/3;<br>O: 0/3;<br>T: 2/3	C: 0/3;<br>O: 1/3;<br>T: 1/3	T: 0/3
Zhejiang	Wenzhou	T: 0/12	T: 0/12	C: 0/12;<br>O: 1/12;<br>T: 1/12

*T: total number of assayed animals.

Table S4. Prevalences of *mcr* genes in cloacal (C) and oropharyngeal (O) swabs in geese.

Province	City	Positive /total samples		
		mcr-1	***mcr-2***	***mcr-3***
Henan	Anyang	C: 6/7;<br>O: 2/7;<br>T*: 6/7	T: 0/7	T: 0/7
Inner Mongolia	Ulanqab	T: 0/5	T: 0/5	T: 0/5
Jiangsu	Yangzhou	C: 7/9;<br>O: 5/9;<br>T: 8/9	T: 0/9	C: 4/9;<br>O: 7/9;<br>T: 9/9
Jiangxi	Xingan	C: 6/9;<br>O: 5/9;<br>T: 8/9	C: 2/9;<br>O: 3/9;<br>T: 4/9	T: 0/9
Shandong	Liaocheng	T: 0/8	T: 0/8	T: 0/8
Shanghai	Shanghai	C: 52/70;<br>O: 36/70;<br>T: 55/70	C: 1/70;<br>O: 1/70;<br>T: 2/70	C: 0/70;<br>O: 4/70;<br>T: 4/70
Zhejiang	Wenzhou	C: 0/1;<br>O: 1/1;<br>T: 1/1	T: 0/1	T: 0/1

*T: total number of assayed animals.

Table S5. Prevalences of *mcr* genes in cloacal (C) and oropharyngeal (O) swabs of pigeons.

Province	City	Positive /total samples		
		mcr-1	***mcr-2***	***mcr-3***
Gansu	Jingyuan	T*: 0/13	T: 0/13	T: 0/13
Hebei	Shijiazhuang	T: 0/34	T: 0/34	T: 0/34
Jiangsu	Yangzhou	C: 11/25;<br>O: 6/10;<br>T: 5/25	T: 0/25	C: 2/25;<br>O: 3/10;<br>T: 5/25
	Yixing	C: 0/21;<br>O: 6/20;<br>T: 6/21	T: 0/21	T: 0/21
Liaoning	Jinzhou	C: 1/6;<br>O: 2/6;<br>T: 2/6	T: 0/6	T: 0/6

*T: total number of assayed animals.

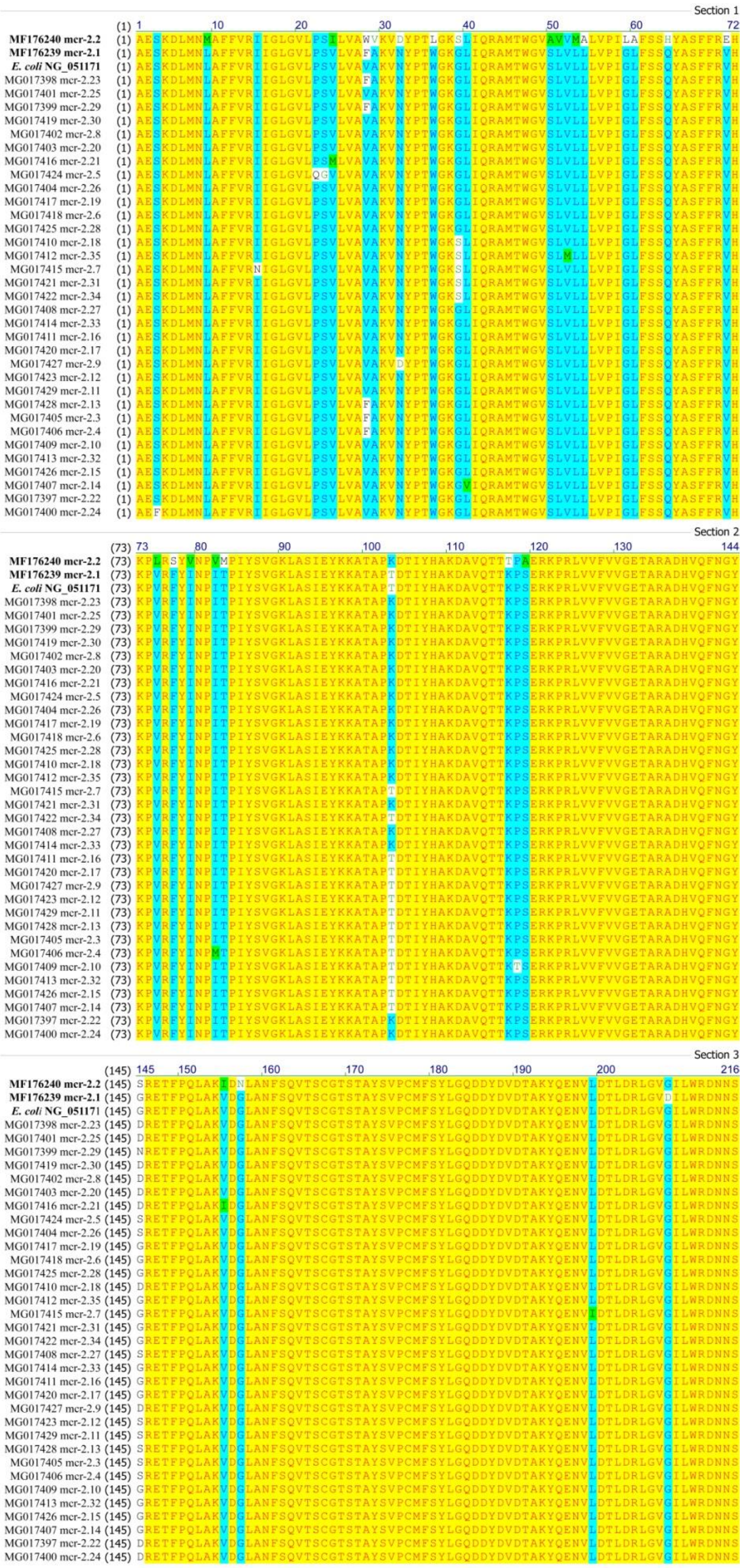

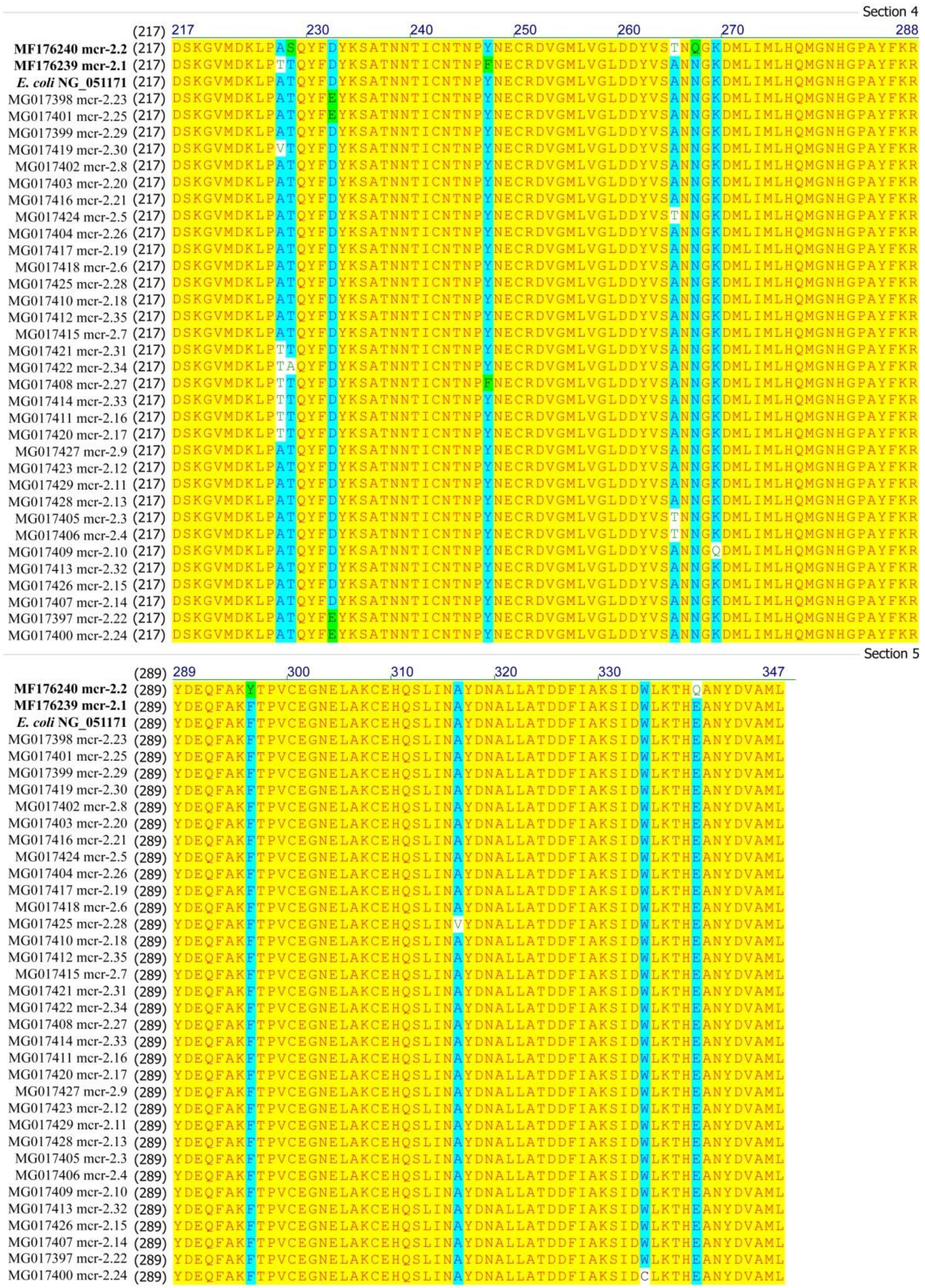

Figure S1. Alignment of amino acid sequences for *mcr-2* gene (347-aa). Amino acids that are identical for the sequences amplified in this study (MG017397-MG017429) and

reference (NG_051171, MF176240, MF176239) from GenBank are highlighted in yellow while those that vary between species are highlighted with blue, green or white.

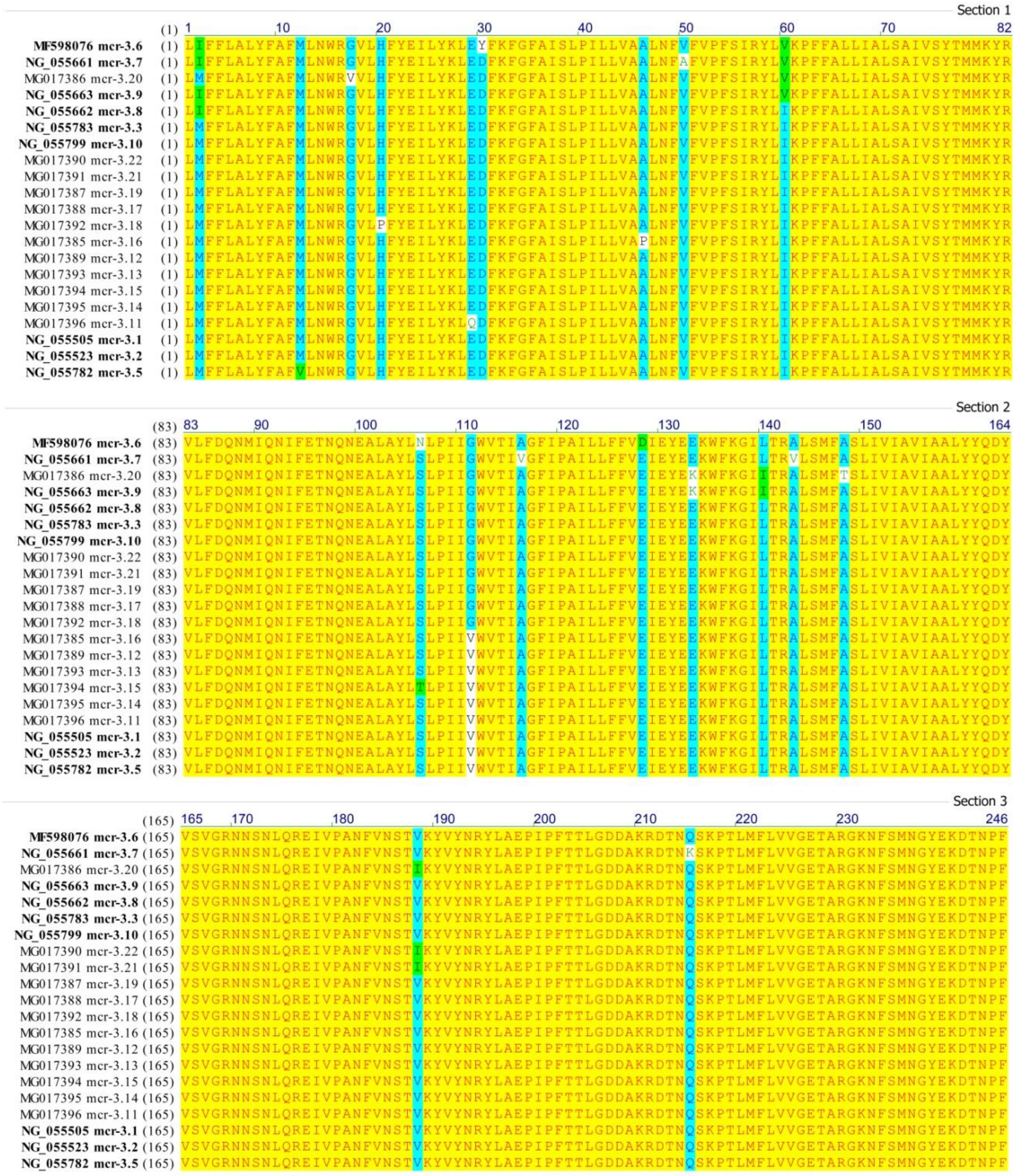

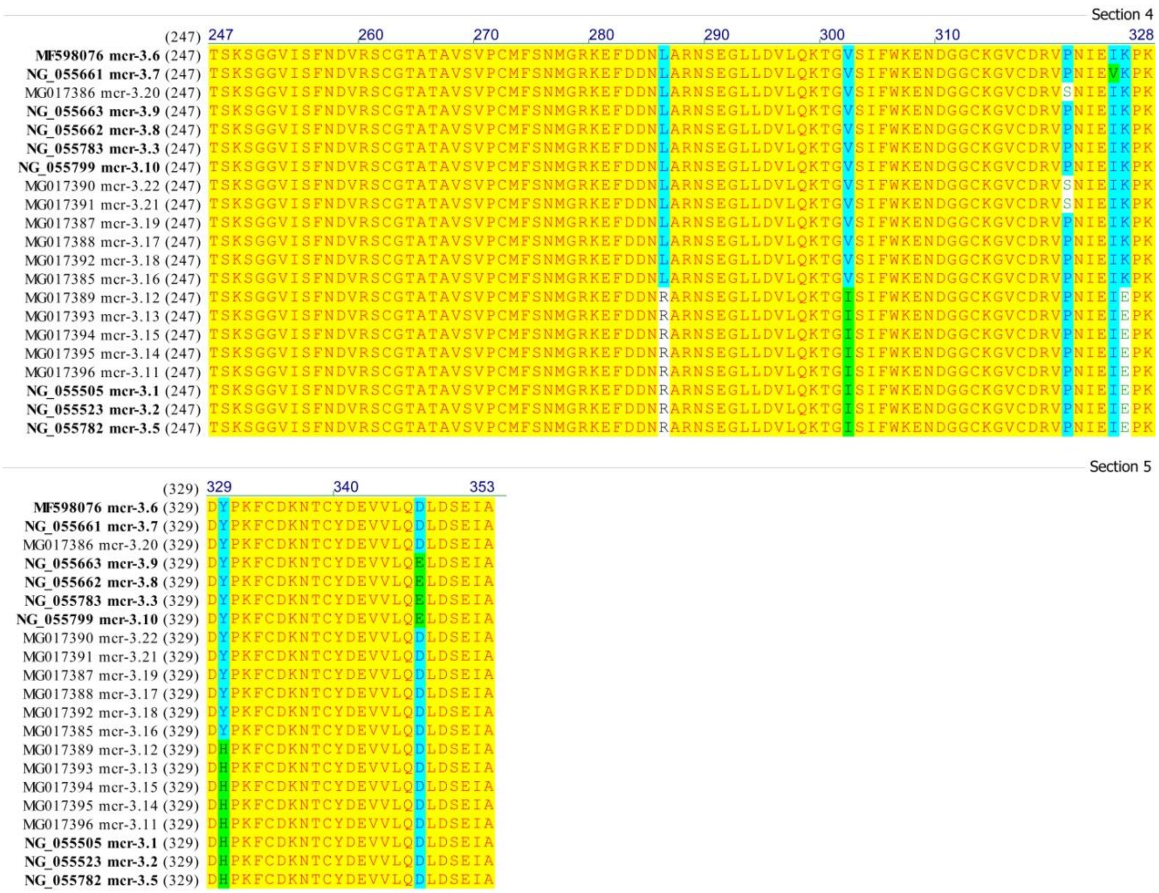

Figure S2. Alignment of amino acid sequences for *mcr-3* gene (353-aa). Amino acids that are identical for the sequences amplified in this study (MG017385-MG017396) and reference (*Escherichia coli* NG_055505) from GenBank are highlighted in yellow while those that vary between species are highlighted with blue or white.
